# Cardiovascular risk profiles in people living with HIV taking antiretroviral therapy in rural Lesotho: Findings from the PRECARIF study

**DOI:** 10.4102/sajhivmed.v27i1.1779

**Published:** 2026-03-11

**Authors:** Moshao A. Makhele, Anna Klicpera, Frédérique Chammartin, Jennifer A. Brown, Philipp Wagner, Bataung Letuma, Moneuoa Majonothane, Niklaus D. Labhardt

**Affiliations:** 1 Division of Clinical Epidemiology, University of Basel, Basel, Switzerland; 2 SolidarMed, Partnerships for Health, Maseru, Lesotho

**Keywords:** cardiovascular risk factors, people with HIV, chronic diseases, arterial hypertension, diabetes mellitus, dyslipidaemia, Framingham risk score, Lesotho

## Abstract

**Background:**

With antiretroviral therapy (ART), people living with HIV (PLHIV) have near-normal life expectancies. As PLHIV get older, diagnosing and managing cardiovascular diseases (CVDs) becomes important. Data on the prevalence of CVD risk factors in PLHIV in Southern Africa remain limited.

**Objectives:**

To determine the prevalence of cardiovascular risk factors among PLHIV receiving ART in Lesotho, Southern Africa.

**Method:**

We prospectively enrolled PLHIV who were receiving ART and attending care at five healthcare facilities in Lesotho between 04 June 2024 and 28 October 2024. Demographic information, medical history, blood pressure, blood glucose, lipid profile, and body-mass-index were measured, and lifestyle risk factors data (tobacco use, alcohol intake, physical activity, diet) were collected. The Framingham risk score (FRS) estimated the 10-year CVD risk.

**Results:**

Of 343 participants, 66.2% were women, and the median age was 50 years (interquartile range 41–59 years old). The prevalence of elevated blood pressure was 49.3%, diabetes mellitus 9.6%, dyslipidaemia 70.6%, and overweight or obesity 62.7%. Tobacco use was reported by 25.7% and alcohol use by 53.6%, of whom 20.1% were heavy drinkers. Of the 343, 89.8% walked or cycled ≥ 10 min daily to commute, 40.2% reported extra salt intake, and 37.0% ate a balanced meal at most once a week. Based on the FRS, 17.6% had a medium and 12.5% a high 10-year CVD risk.

**Conclusion:**

Cardiovascular risk factors are highly prevalent among PLHIV on ART in Lesotho, with nearly one-third at medium or high 10-year CVD risk. These findings support integration of CVD risk management into HIV care in Lesotho.

**What this study adds:** This study provides novel, locally relevant data on cardiovascular risk among people with HIV in Lesotho. The data strengthen the case for integrating cardiovascular risk management into HIV programmes in Lesotho.

## Introduction

The scale-up of antiretroviral therapy (ART) has substantially improved survival among people living with HIV (PLHIV), leading to a growing population ageing with chronic HIV infection and long-term ART exposure.^1^ As HIV/AIDS-related morbidity and mortality have declined, non-communicable diseases (NCDs) – defined as chronic conditions of long duration and slow progression not caused by infection – have emerged as an important component of morbidity among PLHIV.^2^

Globally, NCDs account for the majority of deaths, with cardiovascular diseases (CVDs) representing the largest share.^3,4,5,6^ In sub-Saharan Africa, the burden of NCDs is increasing, driven largely by a rising prevalence of modifiable cardiovascular risk factors such as hypertension, diabetes, dyslipidaemia, obesity, tobacco use, harmful alcohol consumption, physical inactivity, and unhealthy diets.^7,8,9^ Although communicable diseases remain dominant, and absolute CVD event rates are currently lower than in high-income settings, demographic transitions and improved survival are expected to progressively shift disease patterns over time.

People living with HIV may be particularly vulnerable to cardiometabolic abnormalities resulting from a combination of chronic HIV-associated inflammation, metabolic comorbidities, long-term ART exposure, and lifestyle factors.^10,11,12,13^ Studies from southern and eastern Africa have reported a substantial and increasing prevalence of cardiovascular risk factors among PLHIV, including hypertension prevalence of approximately 15% – 30%, alongside variable levels of dyslipidaemia and obesity.^14,15^ However, evidence on overall cardiovascular risk profiles among PLHIV – especially from rural and resource-limited settings in southern Africa – remains limited.

Importantly, while PLHIV in sub-Saharan Africa appear to have a higher relative risk of CVD compared with people without HIV, available evidence suggests that atherosclerotic CVD (ASCVD) currently contributes only a small proportion to the overall NCD burden in the region.^16,17,18^ Characterising cardiometabolic risk profiles among PLHIV may therefore help contextualise emerging vulnerabilities and support integration of HIV and NCD care, rather than implying an immediate need for ASCVD-focused interventions.

Lesotho, which has one of the highest HIV prevalence rates globally, provides an important setting to examine cardiometabolic health among PLHIV in a rural, resource-limited context. The Prevalence of Cardiovascular Risk Factors (PRECARIF) study aimed: (1) to describe the burden of cardiometabolic risk factors and lifestyle-related behaviours among PLHIV in rural Lesotho; and (2) to estimate 10-year ASCVD risk using the Framingham Risk Score as an exploratory risk stratification tool. The analysis focused on modifiable determinants including elevated blood pressure (BP), diabetes, dyslipidaemia, obesity, and tobacco and alcohol use.

## Research methods and design

### Study design, setting and population

We conducted a cross-sectional, non-experimental study to assess the prevalence of cardiovascular risk factors such as elevated BP, diabetes mellitus, dyslipidaemia, obesity, tobacco use, alcohol consumption, physical inactivity, and unhealthy diet among PLHIV attending routine nurse-led ART clinics in north-eastern Lesotho. Between 04 June 2024 and 28 October 2024, we recruited 343 ambulatory adult PLHIV (≥ 18 years old) attending scheduled HIV care and treatment visits at three hospital-based ART clinics and two primary-care health centres in Mokhotlong and Butha Buthe districts (Table 1-A2). All sites functioned as outpatient clinics; no hospitalised patients were included. Participants were selected through systematic random sampling, where every third client presenting for a routine ART visit was approached for study participation.

### Data collection

For each participant, we extracted demographic and clinical information – including sex, date of birth, ethnicity, educational level, and ART history – from medical records. We then verified the information via brief interviews. We assessed lifestyle factors (tobacco use, alcohol use, physical activity, and diet) and socioeconomic status using a structured questionnaire based on the Demographic and Health Surveys.^19^ Additionally, participants were asked whether either of their biological parents had ever been diagnosed with a cardiovascular condition, to assess family history of CVD.

Blood pressure was recorded in the seated position using a calibrated sphygmomanometer; three readings were taken per participant, with the average of the second and third readings used for analysis. Random capillary blood glucose (RBG) was measured using an Accu-Chek^®^ Instant Blood Glucose Meter (Roche Diabetes Care Middle East, FZCO Dubai). Participants with RBG ≥ 5.6mmol/L underwent point–of–care haemoglobin A1c (HbA1c) testing with A1CNow^®^+ SelfCheck (PTS Diagnostics, Whitestown, Indiana, United States). Serum creatinine was assessed with the StatSensor Xpress™ Creatinine test (Nova Biomedical, Waltham and Billerica, Massachusetts, United States), and urine albumin–to–creatinine ratio was determined via a CLINITEK Status^®^+ Analyzer (Siemens Healthineers AG, Forchheim, Germany).

Total cholesterol (TC), high-density lipoprotein (HDL–C), low–density lipoprotein (LDL–C), triglycerides, non–HDL cholesterol, and cholesterol/HDL-C ratio were measured on the Afinion™ 2 Analyzer (Abbott Laboratories, Abbott Park, Illinois, United States). We measured height and weight to calculate body mass index (BMI).

Finally, we estimated each participant’s 10-year cardiovascular risk using the sex–specific Framingham risk score (FRS) algorithm,^20^ which integrates age, sex, smoking status, TC, HDL–C, presence of diabetes, systolic BP, and antihypertensive treatment. All data were entered into the Research Electronic Data Capture (REDCap) database, which we used for data collection and management.

To estimate household wealth of participants, the household wealth questionnaire of the Demographic and Health Surveys^19^ was used. Tobacco use was defined as anyone using tobacco products, whether cigarettes, snuff (common in Lesotho), or other nicotine-containing products, in the past year. Alcohol use was defined as intake of any alcoholic products, which was further quantified, with heavy drinking defined for men as consuming five or more drinks on any day or 15 or more drinks per week, and for women as consuming four or more drinks on any day or eight or more drinks per week. Use of extra salt was defined as the practice of adding salt at the table to food that had already been cooked. Physical activity was defined as either walking or cycling for at least 10 continuous minutes in a day or, on a typical day, participating in vigorous-intensity sport, fitness, or recreational activities.

Elevated BP was defined as systolic BP (SBP) ≥ 140 mmHg and/or diastolic BP (DBP) ≥ 90 mmHg (mean of last two readings). Hypertension was graded as per European Society of Cardiology guidelines (Grade 1: SBP 140 mmHg – 159 mmHg and/or DBP 90 mmHg – 99 mmHg; Grade 2: SBP 160 mmHg – 179 mmHg and/or DBP 100 mmHg – 109 mmHg; Grade 3: SBP ≥ 180 mmHg and/or DBP ≥ 110 mmHg).^21^ Dyslipidaemia was defined as any of the following: elevated total cholesterol (≥ 5.2 mmol/L), abnormal LDL-C (≥ 4.1 mmol/L), abnormal HDL-C (< 1.0 mmol/L in men or < 1.3 mmol/L in women), or hypertriglyceridaemia (> 1.7 mmol/L).^22,23,24^ Diabetes mellitus was defined as current use of antidiabetic medication, or RBG ≥ 5.6 mmol/L with confirmatory HbA1c ≥ 6.5%. Normal RBG is defined as < 5.6 mmol/L).

Body mass index was categorised as underweight (< 18.5 kg/m^2^), normal weight (18.5 kg/m^2^ – 24.9 kg/m^2^), overweight (25.0 kg/m^2^ – 29.9 kg/m^2^), or obese (≥ 30.0 kg/m^2^). The FRS was categorised as low (< 10%), medium (10% – 19%), or high (≥ 20%) 10-year risk of a major cardiovascular event.

### Statistical analysis

Descriptive statistics were used to summarise the characteristics of the participants, by median (with interquartile range [IQR]) for continuous variables, and absolute and relative frequency for categorical variables. Prevalence was estimated as the proportion of individuals meeting the case definition among the total study population, and 95% confidence intervals were calculated using binomial methods to quantify the precision of the estimates.

A sample size of 323 was calculated using the single proportion formula^25^ based on an expected hypertension prevalence of 27% among participants, with a 95% confidence level and a precision of ± 5%. Arterial hypertension was selected as the reference cardiovascular risk factor because of its common occurrence in the population. Statistical analyses were conducted using Stata (version 16.1, StataCorp LLC, College Station, Texas, United States).

### Ethical considerations

The PRECARIF study was approved by the National Health Research Ethics Committee (NH-REC) of the Ministry of Health, Lesotho (REF:ID70-2024). All study participants provided written informed consent. Data were only accessible to authorised personnel who required the data to fulfil their duties within the scope of the research project. On the case report forms and other project-specific documents, participants are only identified by a unique participant number. Study participants received a financial reimbursement of LSL 50 for their time.

## Results

### Sociodemographic and clinical characteristics of the study participants

Overall, 343 participants were enrolled; their sociodemographic and clinical characteristics, as well as lifestyle risk factors, stratified by sex, are shown in Table 1. The median age was 50 years (IQR 41–59), and 227 (66.2%) were women. Most participants were between 45–60 years old (43.1%), followed by 30–44 years old (32.4%). A greater proportion of men compared to women were aged ≥ 60 years (*n* = 35 [30.2%] vs *n* = 38 [16.7%]). Of the participants, 131 (38.2%) reported a family history of CVD. The majority, 320 (93.3%), were taking tenofovir/lamivudine/dolutegravir (TDF/3TC/DTG) for ART. Most participants had been on ART for either 5–10 years (*n* = 131; 38.2%) or 11–15 years (*n* = 108; 31.5%). Most participants had a high school (*n* = 151; 44.0%) or primary school education (*n* = 147; 42.9%).

**TABLE 1:** Sociodemographic, clinical characteristics, and behavioural risk factors.

Characteristics	Men (*n* = 116, 33.8%)				Women (*n* = 227, 66.2%)				Total (*N* = 343, 100%)			
	Median	IQR	*n*	%	Median	IQR	*n*	%	Median	IQR	*n*	%
**Age (years)**	52	42–62	-	-	48	41–58	-	-	50	41–59	-	-
≤ 29	-	-	3	2.6	-	-	8	3.5	-	-	11	3.2
30–44	-	-	32	27.6	-	-	79	34.8	-	-	111	32.4
45–60	-	-	46	39.7	-	-	102	44.9	-	-	148	43.1
> 60	-	-	35	30.2	-	-	38	16.7	-	-	73	21.3
**Level of education**												
Primary school	-	-	53	45.7	-	-	94	41.4	-	-	147	42.9
High school	-	-	44	37.9	-	-	107	47.1	-	-	151	44.0
Tertiary	-	-	7	6.0	-	-	19	8.4	-	-	26	7.6
None	-	-	10	8.6	-	-	2	0.9	-	-	12	3.5
Missing	-	-	2	1.7	-	-	5	2.2	-	-	7	2.0
**ART regimen of participants**												
TDF/3TC/DTG	-	-	108	93	-	-	212	93.4	-	-	320	93.3
ABC/3TC/DTG	-	-	6	5.2	-	-	13	5.7	-	-	19	5.5
AZT/3TC/DTG	-	-	2	1.7	-	-	1	0.4	-	-	3	0.9
ABC/3TC/LPVr	-	-	0	0.0	-	-	1	0.4	-	-	1	0.3
**Time on ART (years)**												
< 5	-	-	21	18.1	-	-	33	14.5	-	-	54	15.7
5–10	-	-	43	37.1	-	-	88	38.8	-	-	131	38.2
11–15	-	-	38	32.8	-	-	70	30.8	-	-	108	31.5
> 15	-	-	14	12.1	-	-	36	15.9	-	-	50	14.6
**Use of tobacco**												
Yes	-	-	45	38.8	-	-	43	18.9	-	-	88	25.7
**Alcohol consumption**												
Yes	-	-	77	66.4	-	-	107	47.1	-	-	184	53.6
Heavy drinking	-	-	40	34.5	-	-	29	12.8	-	-	69	20.1
**Use of extra salt on food (at the table)**												
Yes	-	-	65	56.0	-	-	73	32.3	-	-	138	40.2
Regularly	-	-	30	25.9	-	-	21	9.3	-	-	51	14.9
**Balanced meal**												
Once a week or less often	-	-	44	37.9	-	-	83	36.6	-	-	127	37.0
**Exercise**												
Walking or cycling at least for 10 min to commute	-	-	106	91.4	-	-	202	89.0	-	-	308	89.8
Vigorous intensity sports, fitness, recreational activities	-	-	23	20.0	-	-	37	16.3	-	-	60	17.5

ART, antiretroviral; TDF/3TC/DTG, tenofovir disoproxil fumarate/lamivudine/dolutegravir; ABC/3TC/DTG, abacavir/lamivudine/dolutegravir; AZT/3TC/DTG, zidovudine/lamivudine/dolutegravir; ABC/3TC/LPVr, abacavir/lamivudine/lopinavir/ritonavir; IQR, interquartile range.

### Lifestyle risk factors

Lifestyle risk factors linked to cardiovascular risk, such as alcohol consumption (*n* = 77, 66.4%), tobacco use (*n* = 45, 38.8%), and added salt intake (*n* = 65, 56.0%) were more common among men, compared with alcohol consumption (*n* = 107,47.1%), tobacco use (*n* = 43, 18.9%), and added salt intake (*n* = 73, 32.3%) among women. Overall, 308 participants (89.8%) reported walking as a form of commuting, and 60 participants (17.5%) engaged in regular vigorous activities such as sports, structured fitness, or recreational exercise.

### Prevalence of cardiovascular risk factors and laboratory findings

The clinical characteristics and laboratory findings of the participants are presented in Table 2. The overall prevalence of elevated BP was 49.3% (*n* = 167; 95%CI: 43.3% – 54.1%). The median SBP was 129 mmHg (IQR 118–140), and the DBP was 87 mmHg (IQR 80–93). Overall, half of the participants (50.7%; *n* = 172; 95%CI: 45.4% – 56.1%) had normal BP, while 28.0% (*n* = 95; 95%CI: 23.5% – 33.1%) had grade 1 hypertension, 14.5% (*n* = 49; 95%CI: 11.1% – 18.6%) had grade 2 hypertension, and 6.8% (*n* = 23; 95%CI: 4.54% – 10.0%) had grade 3 hypertension. Grade 3 hypertension was slightly more common in women 11 (9.5%) than men 12 (5.3%), although not statistically significant.

**TABLE 2:** Clinical and laboratory findings and cardiovascular risk factors.

Characteristics	Men (*n* = 116, 33.8%)				Women (*n* = 227, 66.2%)				Total (*N* = 343, 100%)			
	Median	IQR	*n*	%	Median	IQR	*n*	%	Median	IQR	*n*	%
**Hypertension**												
SBP (mmHg)	125.0	118.00–140.00	-	-	129.0	118.0–140.0	-	-	129.0	118.0–140.0	-	-
DBP (mmHg)	88.0	80.00–92.00	-	-	86.0	80.0–95.0	-	-	87.0	80.0–93.0	-	-
Normal BP	-	-	61	53.0	-	-	111	49.6	-	-	172	50.7
Grade 1 Hypertention	-	-	30	26.1	-	-	65	29.0	-	-	95	28.0
Grade 2 Hypertention	-	-	13	11.3	-	-	36	16.1	-	-	49	14.5
Grade 3 Hypertention	-	-	11	9.6	-	-	12	5.4	-	-	23	6.8
Missing	-	-	1	-	-	-	3	-	-	-	4	1.2
**Blood glucose**												
RBG (mmol/L)	5.5	4.90–6.45	-	-	5.7	5.2–6.8	-	-	5.6	5.0–6.8	-	-
< 5.6 mmol/L	-	-	62	53.5	-	-	106	46.7	-	-	168	50.0
5.6 mmol/L – 6.9 mmol/L	-	-	34	29.3	-	-	67	29.5	-	-	101	29.5
7.0 mmol/L – 11.1 mmol/L	-	-	18	15.5	-	-	42	18.5	-	-	60	17.5
≥ 11.1 mmol/L	-	-	2	1.7	-	-	12	5.3	-	-	14	4.1
**HbA1c and known diabetes**												
HbA1c ≥ 6.5%	-	-	0	0.0	-	-	9	8.2	-	-	9	5.4
Known diabetes mellitus	-	-	7	6.0	-	-	17	7.5	-	-	24	7.0
**Family history of cardiovascular disease**												
Positive family history	-	-	41	35.3	-	-	90	39.7	-	-	131	38.2
**Anthropometry – body mass index**												
BMI (kg/m^2^)	23.6	20.90–27.40	-	-	28.4	24.6–32.5	-	-	27.2	23.0–31.0	-	-
Underweight	-	-	4	3.5	-	-	1	0.4	-	-	5	1.5
Normal weight	-	-	62	53.5	-	-	61	26.9	-	-	123	35.9
Overweight	-	-	36	31.0	-	-	75	33.0	-	-	111	32.4
Obese	-	-	14	12.1	-	-	90	39.7	-	-	104	30.3
**Lipid abnormalities**												
Dyslipidaemia	-	-	59	50.9	-	-	183	80.6	-	-	183	80.6
Hypertriglyceridaemia	-	-	37	33.9	-	-	85	37.8	-	-	85	37.8
High total cholesterol	-	-	6	5.3	-	-	17	7.6	-	-	17	7.6
Abnormal HDL cholesterol	-	-	34	29.3	-	-	154	67.8	-	-	154	67.8
Abnormal LDL cholesterol	-	-	0	0.0	-	-	3	1.4	-	-	3	1.4
**Framingham risk score**												
Risk category												
Low risk (< 10%)	-	-	55	50.9	-	-	168	79.6	-	-	223	69.9
Intermediate risk (10% – 19%)	-	-	22	22.2	-	-	32	15.2	-	-	56	17.6
High risk (≥ 20%)	-	-	29	26.9	-	-	11	5.2	-	-	40	12.5

BP, blood pressure; BMI, body mass index; DBP, diastolic blood pressure; HbA1c, haemoglobin A1c or glycated hemoglobin; HDL, high-density lipoprotein; IQR, interquartile range; LDL, low-density lipoprotein; RBG, random blood glucose; SBP, systolic blood pressure.

Glycaemic assessment showed a median RBG of 5.6 mmol/L (IQR 5.0–6.8). Half of the participants (50.0%; *n* = 168; 95%CI: 43.7% – 54.3%) had normal RBG. Overall, 5.4% (*n* = 9; 95%CI: 2.8% – 10.1%) of participants had an HbA1c value in the diabetic range. All of the participants (2.6%; *n* = 9; 95%CI 1.2% – 4.9%) who were newly diagnosed with diabetes through the assessments conducted in this study were women. Participants already taking medication for diabetes accounted for 7.0% (*n* = 24; 95%CI: 4.7% – 11.7%). The median BMI was 27.2 kg/m^2^ (IQR 23.0–31.0). Of the participants, 62.7% (*n* = 215; 95%CI: 57.3% – 67.8%) were overweight or obese, with obesity being more frequent in women than in men (*n* = 90; 39.6% vs *n* = 14; 12.1%). Overall, 70.6% (*n* = 242; 95%CI: 65.5% – 75.2%) were found to have dyslipidaemia; 183 (80.6%) women and 59 (50.9%) men. Hypertriglyceridaemia was found in 35.6% (*n* = 122; 95%CI: 31.5% – 41.9%), and elevated total cholesterol in 6.8% (*n* = 23; 95%CI: 4.6% – 10.1%). Abnormal HDL levels were observed in more than half of the participants (54.8%; *n* = 188; 95%CI: 49.5% – 60.0%), including 154 (67.8%) women and 34 (29.3%) men. Elevated LDL cholesterol was uncommon and was observed in only three (1.0%) participants.

**FIGURE 1:**
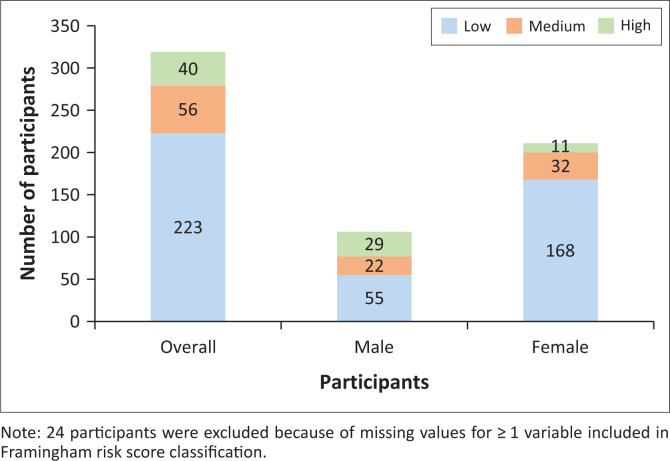
Proportion of study participants by Framingham risk score classification and by sex (*n* = 319).

A family history of CVD was reported by 38.2% (*n* = 131; 95%CI: 33.2% – 43.5%). Figure 1 shows the distribution of CVD risk according to the FRS, stratified by sex. Of the participants, 69.9% (*n* = 223; 95%CI: 64.6% – 74.7%) were at low risk for a cardiovascular event in the next 10 years, 17.6% (*n* = 56; 95%CI: 13.8% – 22.1%) at medium risk, and 12.5% (*n* = 40; 95%CI: 9.3% – 16.7%) at high risk. More men (*n* = 29; 26.9%) than women (*n* = 11; 5.2%) were at high risk. Detailed results stratified by age are provided in Table 1-A1.

## Discussion

The PRECARIF study provides cross-sectional evidence on the prevalence of cardiovascular risk factors and estimated 10-year cardiovascular risk, using the FRS, among 343 adult PLHIV receiving ART in Lesotho. Nearly half of the participants had elevated BP, 9.6% had diabetes mellitus, more than half (62.7%) were overweight or obese, and one in four reported current smoking. Dyslipidaemia was common (70.6%), mainly because of low HDL cholesterol. Overall, nearly one-third of participants had a medium to high 10-year cardiovascular risk. These findings demonstrate that a substantial proportion of PLHIV in a predominantly rural setting face elevated cardiovascular risk, highlighting the importance of considering integrated CVD prevention in this population.

The prevalence of elevated BP in our cohort (48.7%) was considerably higher than the regional average reported for the WHO African Region (27%), and also exceeded community-based estimates from Lesotho (21.6%) and data from Ethiopia (23.8%) among PLHIV.^15,26,27,28,29^ Although direct comparisons should be interpreted cautiously, as we only relied on one singular reading, this suggests a notable burden of hypertension in this population. Factors such as obesity, diet, physical inactivity, and alcohol intake, all common in our study, may contribute, but causal relationships cannot be inferred from this cross-sectional design.

Dyslipidaemia was also common at 70.6%, compared with 76.7% reported in a South African study.^30^ However, the distribution of lipid abnormalities differed between the two populations. In the South African study, elevated triglycerides (62.2%) and total cholesterol (55.2%) were the most common lipid disorders, whereas in our study, the predominant abnormality was low HDL cholesterol (54.8%), followed by hypertriglyceridaemia (36.5%) and elevated total cholesterol (6.8%), with LDL cholesterol abnormalities being rare (< 2%).

These contrasting patterns likely reflect differences in population characteristics, including the impact of HIV and ART in our study, as well as possible contextual differences in diet, lifestyle, and underlying metabolic risk. In the South African study, women had a higher prevalence of dyslipidaemia across all categories, whereas in our population, sex differences were less pronounced. Taken together, these findings highlight that while overall dyslipidaemia prevalence is high in both populations, the specific lipid abnormalities and sex-specific patterns driving this burden vary, emphasising the importance of tailoring prevention and management strategies to the local epidemiological profile. Importantly, in settings such as ours where low HDL-C predominates, traditional LDL-focused lipid-lowering interventions may be insufficient, underscoring the need for broader approaches that incorporate lifestyle modification, metabolic risk control, and HIV-specific care integration.

The overall prevalence of overweight/obesity in our study was 62.7%, with overweight at 32.4%, and obesity at 30.3%. These results are consistent with the 45.4% prevalence reported in a cohort of PLHIV in Botswana.^31^ Consistent with the latter study, we observed higher rates of overweight/obesity among female participants.

The co-existence of multiple risk factors, such as high rates of overweight or obesity (62.7%), diabetes mellitus (9.6%), and dyslipidaemia (70.6%), mirrors findings from other African settings, where PLHIV increasingly develop NCDs.^15,32,33^

Lesotho has undergone significant urbanisation, yet much of the population still experiences food insecurity, contributing to poor diet diversity. Health education and NCD prevention services are limited, and the national health system remains heavily focused on infectious diseases. Lifestyle factors such as tobacco use (25.7%), heavy alcohol intake (20.1%), and high salt consumption (40.2%) were highly prevalent in our sample, reflecting broader behavioural and cultural trends.

Importantly, nearly one-third of participants were at medium or high risk of a 10-year cardiovascular event based on FRS. Although this tool was developed in predominantly Caucasian populations and does not incorporate HIV-related or ART-related factors, it provides a pragmatic estimate of risk and supports prioritising screening and risk reduction efforts in HIV clinics. While recent evidence from the REPRIEVE trial^10^ supports statin use for cardiovascular prevention in selected people with HIV, extrapolation to routine statin therapy in sub-Saharan Africa should be cautious; in our study, the approximately one-third of participants classified as having moderate or high FRS should be prioritised for comprehensive cardiovascular risk assessment and management, rather than universal statin initiation.

In summary, the PRECARIF findings demonstrate the need for integration of CVD screening and management into routine HIV care in Lesotho. Integrated care offers a cost-effective, patient-centred approach, especially in resource-limited settings. For example, in South Africa, the Integrated Chronic Disease Management model has demonstrated success in co-managing HIV, hypertension, and diabetes at the primary care level.^34^ Similarly, in Kenya, pilot programmes integrating CVD risk screening into HIV clinics have improved early diagnosis and treatment initiation for hypertension and diabetes.^35^

### Strengths and limitations

The PRECARIF study adds important evidence on cardiovascular risk among PLHIV in rural Lesotho, a context where data are limited. Strengths include a comprehensive assessment of both clinical and lifestyle risk factors, using standardised protocols, integration of medical records with structured interviews to enhance data accuracy, and application of internationally comparable risk assessment tools.

This study has limitations. First, the cross-sectional design precludes causal inference, and the lack of a control group without HIV limits comparability with the general population. Second, though our BP estimates are based on standardised triplicate measurements averaged following the WHO STEPS protocol, which reduces random variability and white-coat effects relative to single readings, there was no confirmatory BP measurement on a different day. Thus, the reported prevalence of elevated BP in our study may overestimate the true prevalence of hypertension. Third, the FRS has not been validated among people with HIV in Africa or in populations with a low overall incidence of ASCVD. In such settings, the tool may demonstrate suboptimal discrimination and calibration, with a tendency to overestimate absolute cardiovascular risk, which may limit the accuracy of risk estimates in our study population. Fourth, the study population was drawn from PLHIV engaged in care in rural clinics, limiting generalisability to urban populations and to people with HIV not engaged in care. Finally, self-reported lifestyle behaviours, such as alcohol and tobacco use, may be affected by recall or social desirability bias.

## Conclusion

People living with HIV in rural Lesotho bear a considerable burden of cardiovascular risk factors, including elevated BP, overweight or obesity, and metabolic abnormalities. These findings highlight the need to strengthen routine screening for BP, blood glucose, lipid screening and lifestyle risk factors within existing ART services. Integrating such assessments into existing HIV care platforms may facilitate earlier identification and management of cardiometabolic risk and help address the evolving health needs of this population.

## Appendix 1

**TABLE 1-A1:** Clinical and laboratory findings and cardiovascular risk factors by age group.

Characteristic	Age group																			
	≤ 29 years (*n* = 11)				30-44 years (*n* = 111)				45-60 years (*n* = 148)				> 60 years (*n* = 73)				Total (*N* = 343)			
	Median	IQR	*n*	%	Median	IQR	*n*	%	Median	IQR	*n*	%	Median	IQR	*n*	%	Median	IQR	*n*	%
**Blood pressure**																				
SBP	110.0	110.0–125.0	-	-	123.0	115.0–135.0	-	-	130.0	119.0–140.0	-	-	130.0	120.0–140.0	-	-	129.0	118.0–140.0	-	-
DBP	85.0	74.0–90.0	-	-	87.0	80.0–93.0	-	-	88.0	80.0–95.0	-	-	86.0	80.0–90.0	-	-	87.0	80.0–93.0	-	-
Normal BP	-	-	7	63.6	-	-	56	51.4	-	-	73	50.0	-	-	36	49.3	-	-	172	50.7
Grade 1	-	-	3	27.3	-	-	33	30.3	-	-	39	26.7	-	-	20	27.4	-	-	95	28.0
Grade 2	-	-	0	0.0	-	-	14	12.8	-	-	24	16.4	-	-	11	15.1	-	-	49	14.5
Grade 3	-	-	1	9.1	-	-	6	5.5	-	-	10	6.8	-	-	6	8.2	-	-	23	6.8
Missing	-	-	0	0.0	-	-	2	1.8	-	-	2	1.4	-	-	0	0.0	-	-	4	1.2
**BMI**																				
BMI	23.9	21.3–26.0	-	-	26.9	23.3–31.1	-	-	27.7	22.7–32.4	-	-	26.9	22.3–28.3	-	-	27.2	22.9–31.0	-	-
Underweight	-	-	0	0.0	-	-	0	0.0	-	-	3	2.0	-	-	2	2.7	-	-	5	1.5
Normal	-	-	7	63.6	-	-	40	36.0	-	-	49	33.1	-	-	27	37.0	-	-	123	35.9
Overweight	-	-	3	27.3	-	-	37	33.3	-	-	38	25.7	-	-	33	45.2	-	-	111	32.4
Obesity	-	-	1	9.1	-	-	34	30.6	-	-	58	39.2	-	-	11	15.1	-	-	104	30.3
**RBG**																				
RBG	4.9	4.4–5.7	-	-	5.7	5.0–6.8	-	-	5.5	5.2–6.8	-	-	5.8	5.1–6.8	-	-	5.6	5.0–6.8	-	-
< 5.6 mmol/L	-	-	8	72.7	-	-	53	47.7	-	-	75	50.7	-	-	32	43.8	-	-	168	49.0
5.6 mmol/L – 6.9 mmol/L	-	-	3	27.3	-	-	34	30.6	-	-	39	26.4	-	-	25	34.2	-	-	101	29.4
7.0 mmol/L – 11.1 mmol/L	-	-	0	0.0	-	-	19	17.1	-	-	29	19.6	-	-	12	16.4	-	-	60	17.5
> 11.1mmol/L	-	-	0	0.0	-	-	5	4.5	-	-	5	3.4	-	-	4	5.5	-	-	14	4.1
**HbA1c (if RBG ≥ 5.6%)**																				
HbA1c	4.7	0.0–5.1	-	-	5.0	4.8–5.4	-	-	5.1	4.6–5.4	-	-	5.2	4.9–5.6	-	-	5.1	4.7–5.4	-	-
< 6.5%	-	-	6	100	-	-	55	94.8	-	-	63	92.6	-	-	33	97.1	-	-	157	94.6
≥ 6.5%	-	-	0	0.0	-	-	3	5.2	-	-	5	7.4	-	-	1	2.9	-	-	9	5.4
**Dyslipidaemia**																				
Yes	-	-	6	54.6	-	-	86	77.5	-	-	100	67.7	-	-	50	68.5	-	-	242	70.6
**Triglycerides**																				
Hypertriglyceridaemia	-	-	2	22.2	-	-	38	34.9	-	-	55	37.4	-	-	27	39.1	-	-	122	36.5
**Total cholesterol**																				
High cholesterol	-	-	0	0.0	-	-	6	5.5	-	-	9	6.1	-	-	8	11.3	-	-	23	6.8
**HDL cholesterol**																				
Abnormal	-	-	6	54.5	-	-	72	64.9	-	-	79	53.4	-	-	31	42.5	-	-	188	54.8
**LDL cholesterol**																				
Abnormal	-	-	0	0.0	-	-	1	1.0	-	-	1	0.7	-	-	1	1.5	-	-	3	1.0
**Tobacco use**																				
Yes	-	-	1	9.1	-	-	29	26.1	-	-	43	29.1	-	-	15	20.5	-	-	88	25.7
**Use of extra salt on food**																				
Yes	-	-	3	27.3	-	-	50	45.0	-	-	56	37.8	-	-	29	39.7	-	-	138	40.2
**Framingham risk score**																				
Low risk	-	-	0	0.0	-	-	101	94.4	-	-	106	73.1	-	-	16	23.9	-	-	223	69.9
Medium risk	-	-	0	0.0	-	-	2	1.9	-	-	28	19.3	-	-	26	38.8	-	-	56	17.6
High risk	-	-	0	0.0	-	-	4	3.7	-	-	11	7.6	-	-	25	37.3	-	-	40	12.5

BMI, body mass index; BP, blood pressure; CVDs, cardiovascular diseases; SBP, systolic blood pressure; DBP, diastolic blood pressure; HbA1c, haemoglobin A1c (glycated haemoglobin); HDL, high-density lipoprotein; IQR, interquartile range; LDL, low-density lipoprotein; RBG, random blood glucose.

## Appendix 2

**TABLE 1-A2:** Participant distribution by study site and sex.

Facility of enrolment	Male	Female	Total
Butha-Buthe Government Hospital	85	146	231
Mokhotlong Government Hospital	1	55	56
Mapholaneng Health Centre	10	11	21
Seboche Mission Hospital	14	5	19
St Paul Health Centre	6	10	16
**Total**	**116**	**227**	**343**
